# Supplementary figures and images for: Wolbachia infection at least partially rescues the fertility and ovary defects of several new Drosophila melanogaster bag of marbles protein-coding mutants
Source: PLoS Genet. 2023 Oct 23;19(10):e1011009. doi: 10.1371/journal.pgen.1011009 (PMC10621935; doi:10.1371/journal.pgen.1011009)

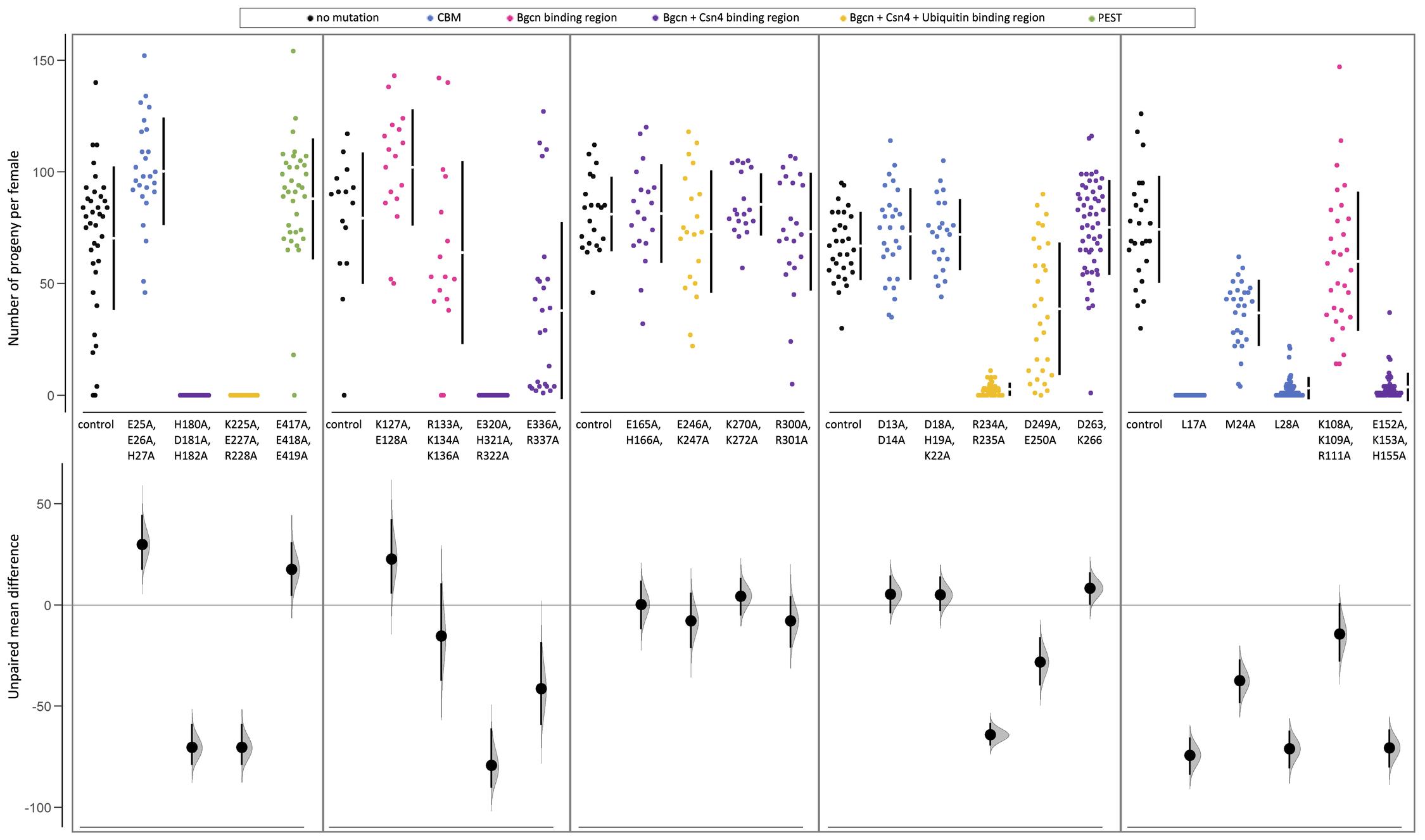

Supplement: S1 Fig — Fertility assays were done in five independent rounds. Below the jitter plots are the resampled bootstrap sampling distributions, with the mean differences represented by the black dots and the 95% confidence intervals represented by the vertical lines. Mean and sample size for each sample are listed in S3 Table. (TIF) [file pgen.1011009.s001.tif]

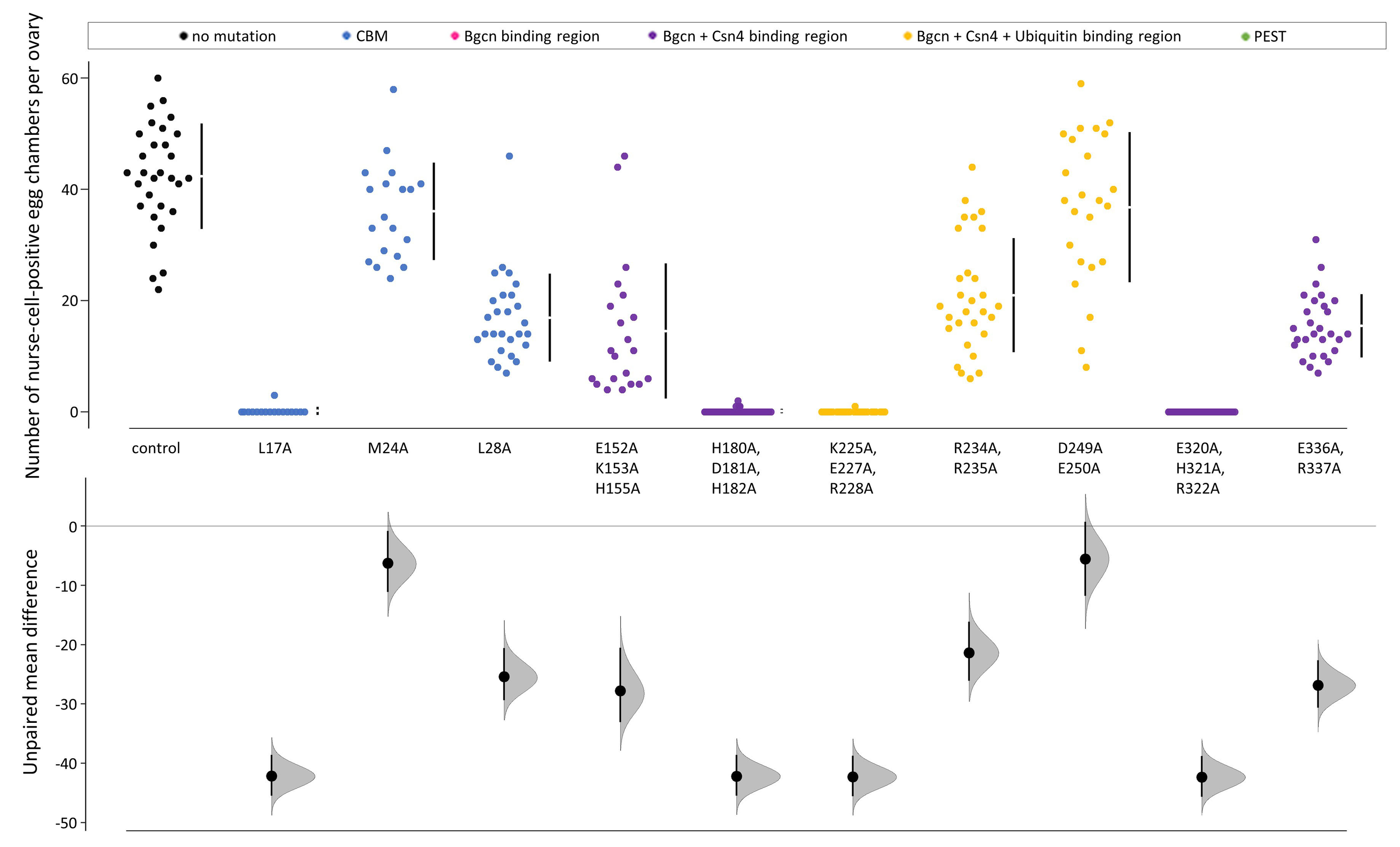

Supplement: S2 Fig — Below the jitter plots are the resampled bootstrap sampling distributions, with the mean differences represented by the black dots and the 95% confidence intervals represented by the vertical lines. Mean and sample size for each sample are listed in S4 Table. (TIF) [file pgen.1011009.s002.tif]

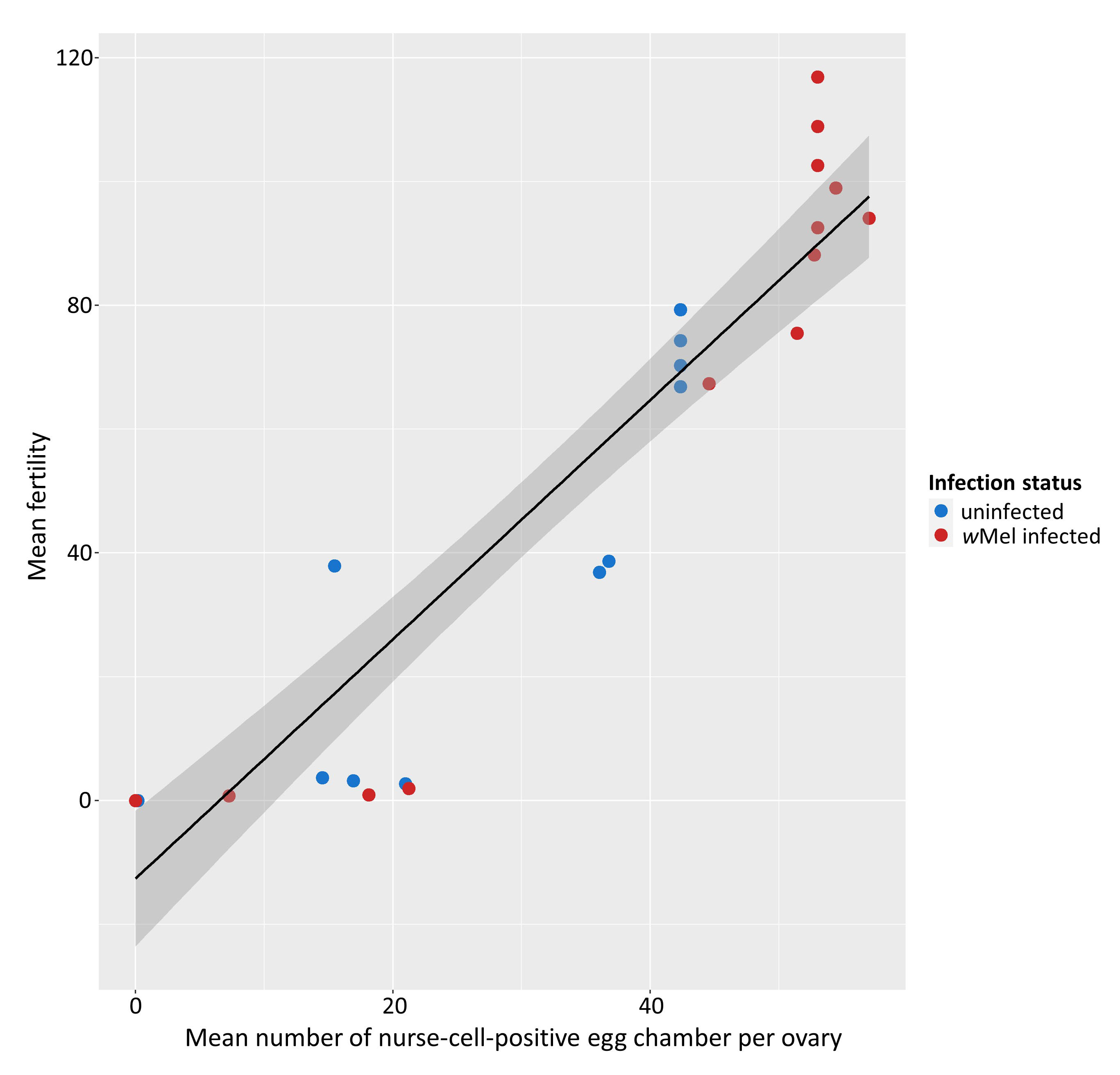

Supplement: S3 Fig — Each point represents the mean data for a fertility defective transgenic bam mutant line, with uninfected data in blue and infected data in red. Kendall’s rank correlation τ = 0.828. (TIF) [file pgen.1011009.s003.tif]

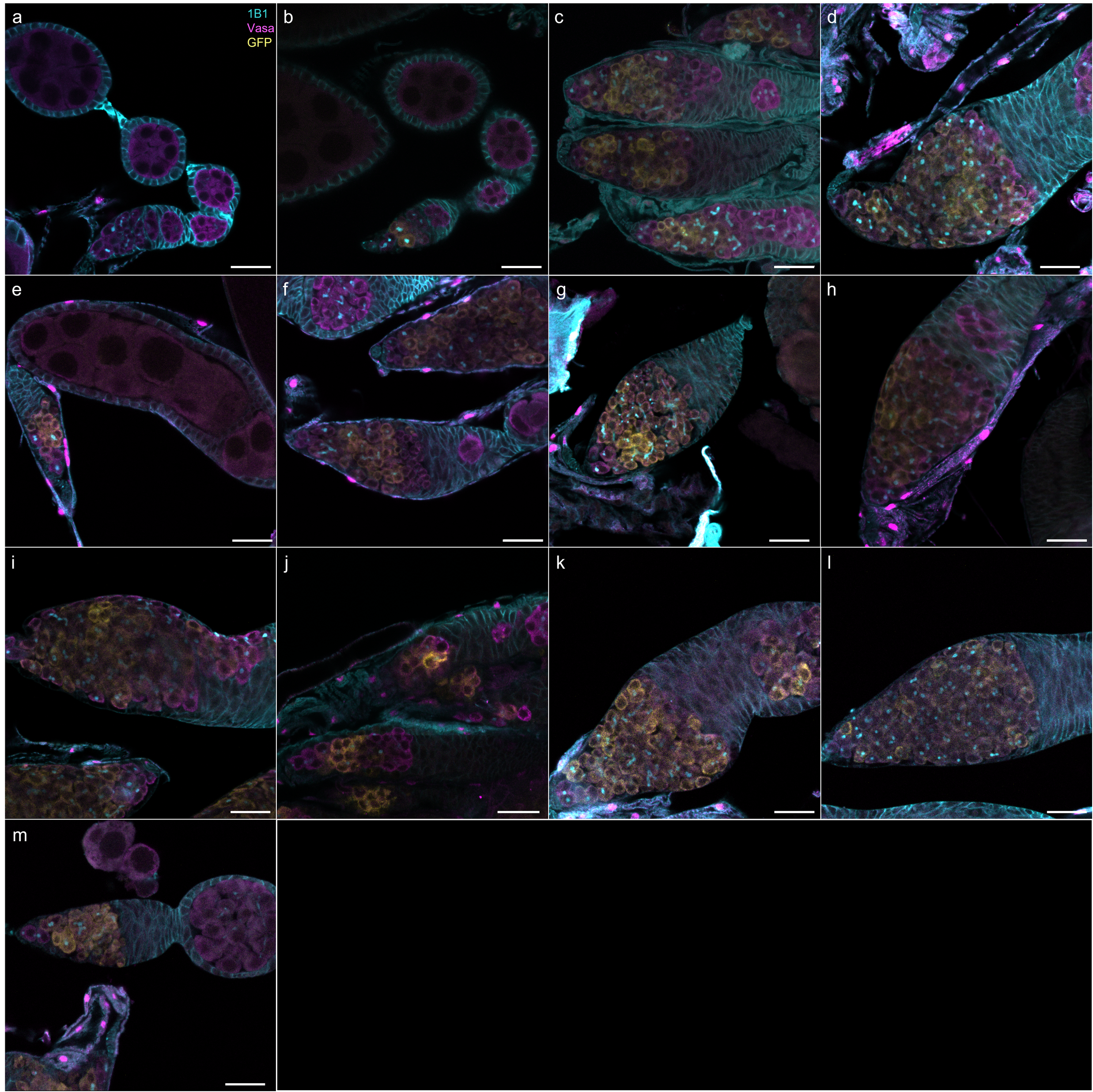

Supplement: S4 Fig — Stained with anti-Hts-1B1 (cyan), anti-Vasa (magenta), and anti-GFP (yellow). (a) CantonS, (b) bam::Venus control, (c) bamL255F::Venus, (d) bamL17A::Venus, (e) bamM24A::Venus, (f) bamL28A::Venus, (g) bamE152A, K153A, H155A::Venus, (h) bamH180A, D181A, H182A:::Venus, (i) bamR234A, R235A::Venus, (j) bamD249A, E250A::Venus, (k) bamK255A, E227A, R228A::Venus, (l) bamE320A, H321A, R322A::Venus, (m) bamE336A, R337A::Venus. Scale bar is 20μM for all images. (TIF) [file pgen.1011009.s004.tif]

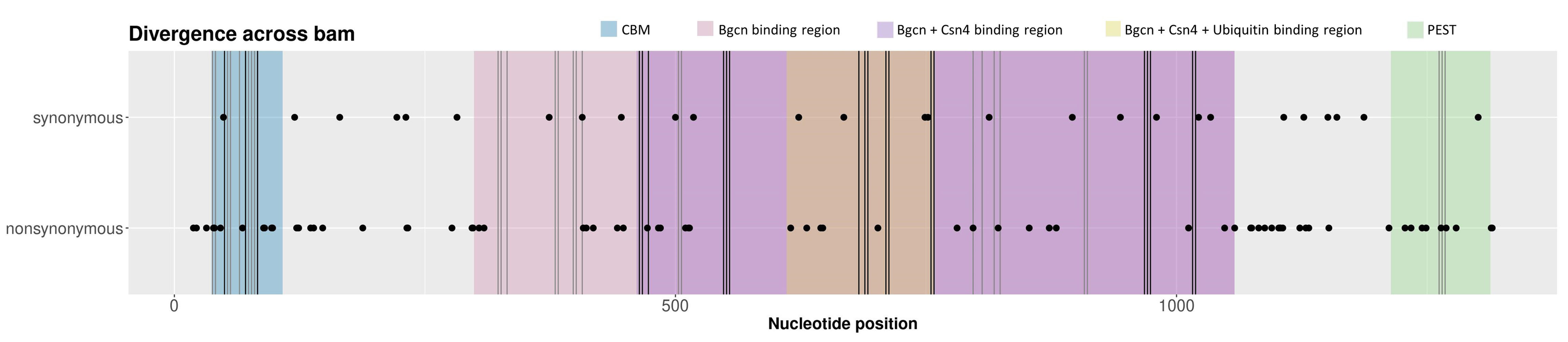

Supplement: S5 Fig — Location of alanine mutations are represented by vertical lines, with fertility defect mutants in black and non-defect mutants in grey. Black dots represent the location of divergent amino acids between D. melanogaster and D. simulans, with synonymous divergences in the top row and nonsynonymous divergences in the bottom row. Background colors correspond to known functional and/or binding regions in D. melanogaster. (TIF) [file pgen.1011009.s005.tif]

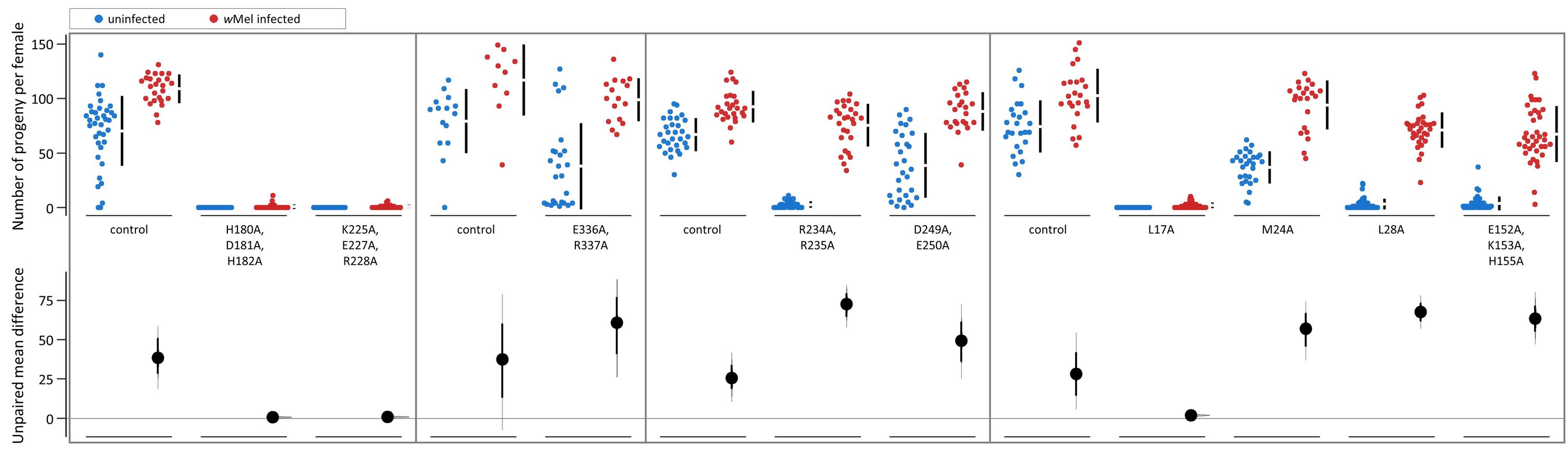

Supplement: S6 Fig — Below the jitter plots are the resampled bootstrap sampling distributions, with the mean differences between the uninfected and infected data represented by the black dots and the 95% confidence intervals represented by the vertical lines. Mean and sample size for each sample are listed in S3 Table. (TIF) [file pgen.1011009.s006.tif]

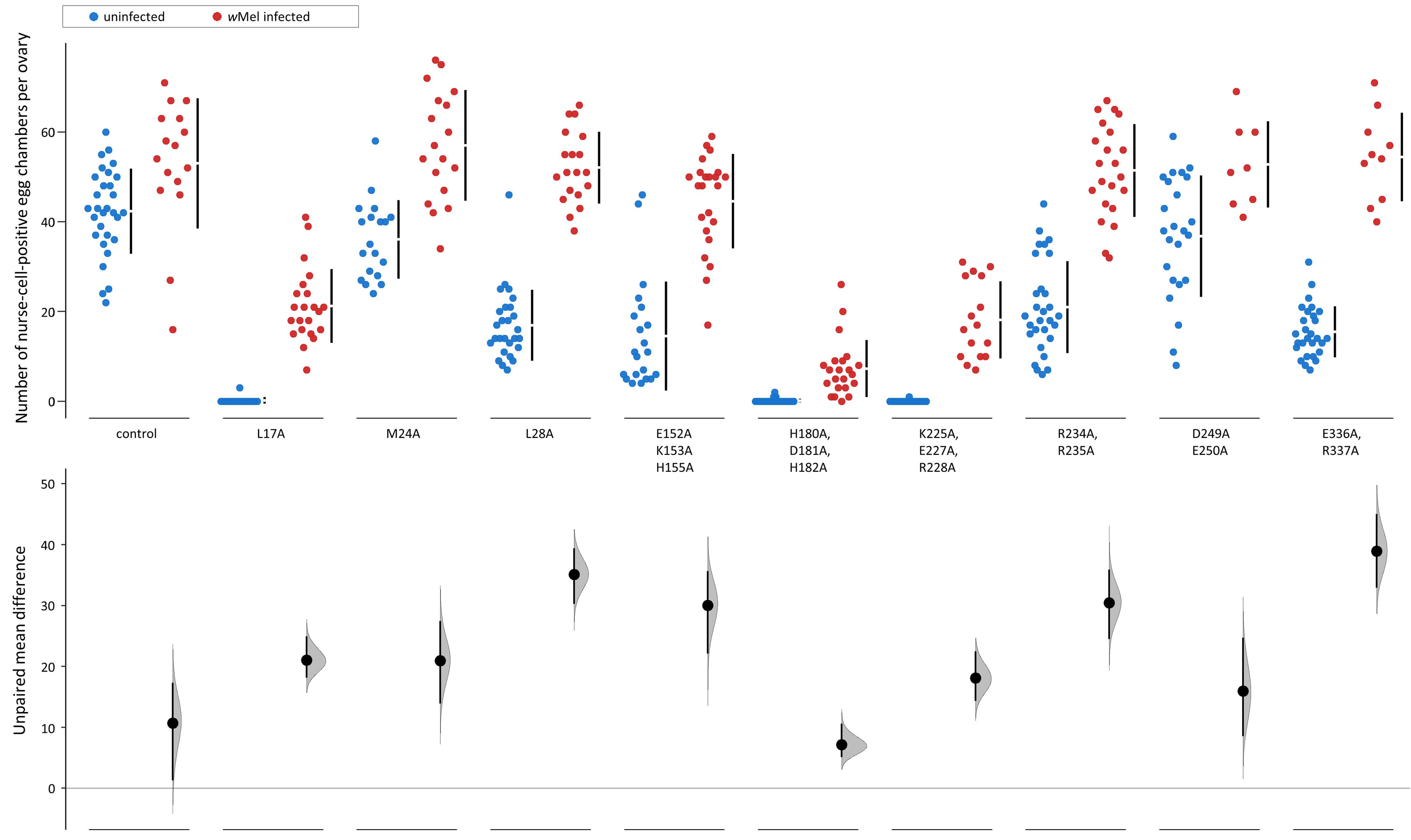

Supplement: S7 Fig — Below the jitter plots are the resampled bootstrap sampling distributions, with the mean differences between the uninfected and infected data represented by the black dots and the 95% confidence intervals represented by the vertical lines. Mean and sample size for each sample are listed in S4 Table. (TIF) [file pgen.1011009.s007.tif]

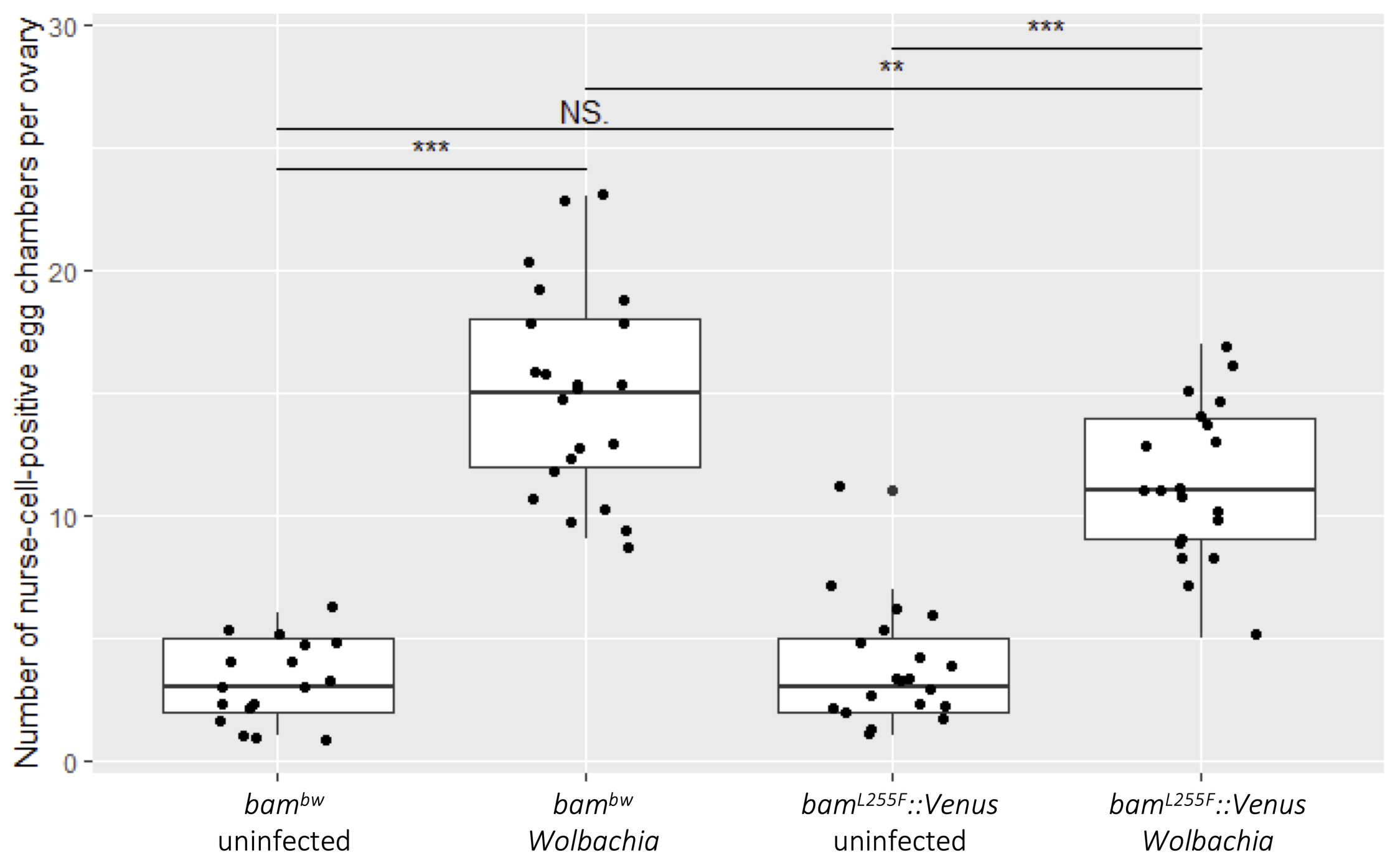

Supplement: S8 Fig — ** = significant at p<0.01; *** = significant at p<0.001; NS = not significant. (TIF) [file pgen.1011009.s008.tif]

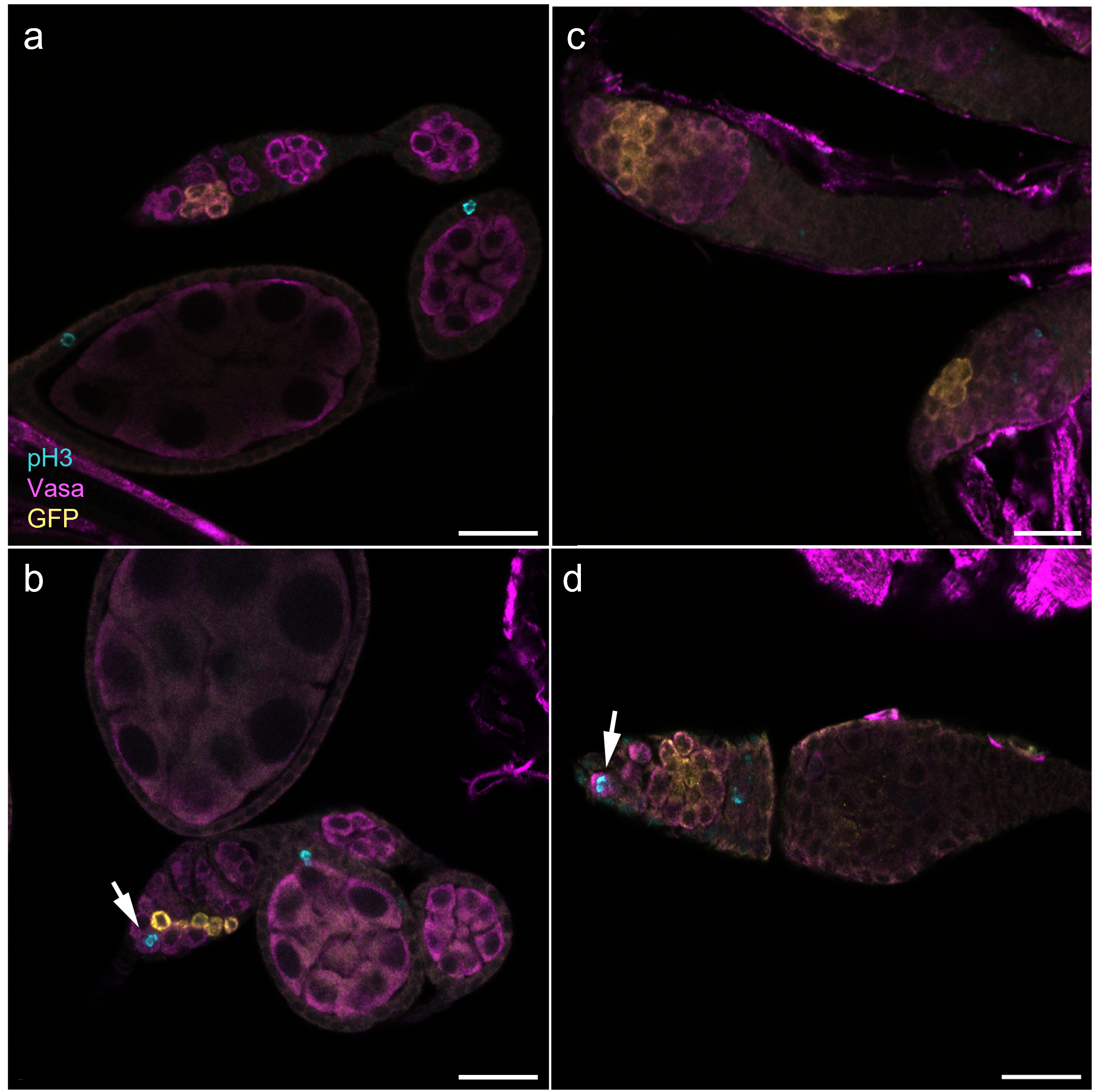

Supplement: S9 Fig — (a) No GSC mitosis and (b) active GSC mitosis in the bam::Venus control germarium. (c) No GSC mitosis and (d) active GSC mitosis in the bamL255F::Venus hypomorphic mutant. (TIF) [file pgen.1011009.s009.tif]

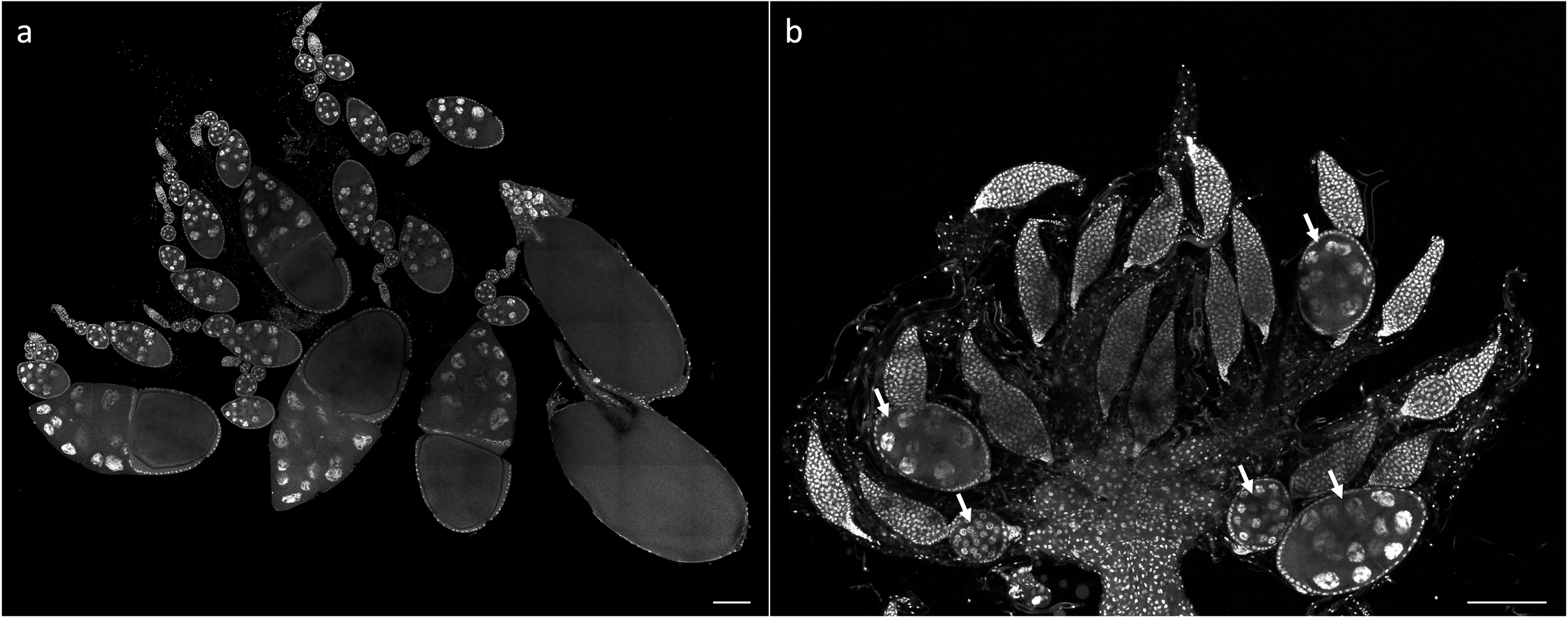

Supplement: S10 Fig — (a) Ovaries of a bam wildtype fly. All egg chambers are results of successful Bam function. (b) Mutant ovaries of a nos-Gal4/+; UAS-bamHMS00029/+ uninfected fly. Arrows indicate nurse-cell-positive egg chambers that represent successful Bam function. Scale bar for both images is 100μM. (TIF) [file pgen.1011009.s010.tif]
