# Supplementary material for: Wolbachia infection at least partially rescues the fertility and ovary defects of several new Drosophila melanogaster bag of marbles protein-coding mutants
Source: PLoS Genet. 2023 Oct 23;19(10):e1011009. doi: 10.1371/journal.pgen.1011009 (PMC10621935; doi:10.1371/journal.pgen.1011009)
Supplement: Attachment — Submitted filename: 7.16.23_Response_to_Reviewers.docx [file pgen.1011009.s017.docx]

**A note from authors:**

Below, the original letter and comments we received are listed in black, italic text. Our responses to the various comments are in purple, preceded by “Response from authors.”

Thank you!

-------------------------------------------------------------------------------------------------------------------------------

Dear Dr Wenzel,

Thank you very much for submitting your Research Article entitled 'Wolbachia rescue of several Drosophila melanogaster bag of marbles protein-coding mutants' to PLOS Genetics.

The manuscript was fully evaluated at the editorial level and by two independent peer reviewers, who are both experts in the field. The reviewers appreciated the attention to an important problem, but raised some substantial concerns about the current manuscript. Based on the reviews, we will not be able to accept this version of the manuscript, but we would be willing to review a much-revised version that incorporates their constructive criticism to better present the current work into a larger biological framework; one reviewer commented that they thought the work as presented was quite narrow. We cannot, of course, promise publication at that time but we will consider the revision in context of these reviews and your revisions.

Should you decide to revise the manuscript for further consideration here, your revisions should address the specific points made by each reviewer. We will also require a detailed list of your responses to the review comments and a description of the changes you have made in the manuscript.

If you decide to revise the manuscript for further consideration at PLOS Genetics, please aim to resubmit within the next 60 days, unless it will take extra time to address the concerns of the reviewers, in which case we would appreciate an expected resubmission date by email to plosgenetics@plos.org.

If present, accompanying reviewer attachments are included with this email; please notify the journal office if any appear to be missing. They will also be available for download from the link below. You can use this link to log into the system when you are ready to submit a revised version, having first consulted our [Submission Checklist](http://journals.plos.org/plosgenetics/s/submit-now#loc-submission-checklist).

To enhance the reproducibility of your results, we recommend that you deposit your laboratory protocols in [protocols.io](http://protocols.io), where a protocol can be assigned its own identifier (DOI) such that it can be cited independently in the future. Additionally, PLOS ONE offers an option to publish peer-reviewed clinical study protocols. Read more information on sharing protocols at<https://plos.org/protocols?utm_medium=editorial-email&utm_source=authorletters&utm_campaign=protocols>

Please be aware that our [data availability policy](http://journals.plos.org/plosgenetics/s/data-availability) requires that all numerical data underlying graphs or summary statistics are included with the submission, and you will need to provide this upon resubmission if not already present. In addition, we do not permit the inclusion of phrases such as "data not shown" or "unpublished results" in manuscripts. All points should be backed up by data provided with the submission.

While revising your submission, please upload your figure files to the [Preflight Analysis and Conversion Engine](http://pace.apexcovantage.com/) (PACE) digital diagnostic tool.  PACE helps ensure that figures meet PLOS requirements. To use PACE, you must first register as a user. Then, login and navigate to the UPLOAD tab, where you will find detailed instructions on how to use the tool. If you encounter any issues or have any questions when using PACE, please email us at figures@plos.org.

PLOS has incorporated [Similarity Check](http://www.crossref.org/crosscheck.html), powered by iThenticate, into its journal-wide submission system in order to screen submitted content for originality before publication. Each PLOS journal undertakes screening on a proportion of submitted articles. You will be contacted if needed following the screening process.

To resubmit, use the link below and 'Revise Submission' in the 'Submissions Needing Revision' folder.

We are sorry that we cannot be more positive about your manuscript at this stage. Please do not hesitate to contact us if you have any concerns or questions.

Yours sincerely,

Harmit S. Malik

Academic Editor

PLOS Genetics

Gregory P. Copenhaver

Editor-in-Chief

PLOS Genetics

-------------------------------------------------------------------------------------------------------------------------------

*Reviewer's Responses to Questions*

***Comments to the Authors:
Please note here if the review is uploaded as an attachment.***

*Reviewer #1: This paper follows previous work from the lab published in 2015 in the same journal. That paper showed Wolbachia infection also partially rescues the sterility of bam hypomorphic mutants. Here they continue this line of research by testing rescue of fertility defects across 22 new bam mutants in which the most compelling data of the paper indicate Wolbachia rescue a variety of mutations that reduce fertility of uninfected mutants in the range of 1-50% fertility reductions, but mostly not above or below. The results also support that no single function stands out for how Wolbachia rescues bam function.

Major comments:

Line 74: The connection between Sxl and bam is an intriguing hypothesis. That would be fun to test!*

**Response from authors:** We agree that this connection is intriguing, which is why we noted it. At the moment, testing is beyond the scope of this paper. We appreciate your enthusiasm on the matter!

*Line 132: The mutants were created with a Venus fluorescent tag. Mention if this is a variable in the experiment that is controlled for or not.*

**Response from authors:** We added text to this Materials and Methods section to state upfront that the Venus fluorescent tag is controlled for in these experiments (L142-L143).  *Line 178: How can it be surmised that a fertility defect does not result from a vigor defect of the mutant (e.g. do mutant females escape from the two mating males they are paired with so that the female cannot lay enough eggs)? For instance, if the males were removed from the assay, maybe some of the mutant females would have no fertility defect. That could significantly change the interpretations. I recognize there might be some wiggle room here about semantically defining fertility and vigor competition with males, but it would be good to clarify these options in the text and use generalist language to reflect their biological differences. The ovary data is not yet convincing yet to firmly conclude the bam mutation only works through impact on ovaries (See below)*

**Response from authors:** Thank you for these comments on the rigor of our experimental results and interpretations. To address your comment, we have since performed the nurse-cell-positive egg chamber experiment for our novel *bam* fertility mutants (L311-L325, Figure 3 and L378-406, Figure 6). This is an experiment in which we quantify the number of egg chambers that have nurse cells, which indicates a successful GSC daughter cell differentiation event. Therefore, this experiment gives us a better reflection of the more immediate consequences of *bam*’s known function in differentiation than the fertility experiments that we have performed. (This is the same assessment that was performed for our *bam* RNAi knockdown experiment that is in this manuscript, as well as had been performed by other members of our lab for their respective papers (see Flores et al. 2015 and Bubnell et al. 2021).)

As *bam* functions early in gametogenesis, we don’t have strong reason to believe it impacts mating behavior. A future pilot study would be great to assess this. Thus, while additional experiments assessing potential vigor defects are possible to do, we believe the assay we chose is most appropriate at this time. We see that our new results generally correlate with our original fertility results (L323-325).

*Line 223+: Does an ovary defect technically and directly translate to a fly fertility defect? The work can provide quantitative data that would support a statistical correlation between the average fly fertility effect versus the average (or %) defect in the ovary to build the case. Currently, single to a few images are presented; good evidence would be quantitative and backed by statistics. This seems important to resolve so the above alternative explanation about vigor defects could be rendered a bit less likely.*

**Response from authors:** See our response above to the reviewer comment for Line 178.

*Line 314+: The mutants without a fly fertility defect would be great to show that ovary cytology is normal and thus correlates well with the fly mutants with normal fertility. Is this available?*

**Response from authors:** Our focus was on the fertility defect mutants and thus we do not have this available. In our manuscript revisions, we chose to focus on the Line 178 and Line 223+ comments for the immediate future.

*Minor comments:

Figure 1: Great figure and good to connect with a similar figure at the end of the article.*

**Response from authors:** We are glad the reviewer likes and appreciates this figure!

*Line 63: For equity and since most articles do not have authors names, restructure the sentence to be agnostic to author names" to avoid unintentional bias.*

**Response from authors:** We edited the sentence to omit authors’ names (L69). Thank you for the advice- we will be mindful of this for future manuscripts.

*Line 271+: The alternative explanation for the widespread effects of the mutations
on fertility is a disruption of protein structure. This can be added here and other spots.*

**Response from authors:** The primary intent of our alanine-scanning approach is to minimize the potential effects on protein structure, as stated in L523-525. However, this reviewer correctly points out that a disruption on protein structure is still a possibility. We have added this point to the Discussion section, where we think it fits best (L531-533).

*Figure 5: Which sites are exposed on the surface versus internal to the protein? A view of the protein surface with color coded sites will help.*

**Response from authors:** We agree that such a view would be extremely interesting, the fact that much of the protein is disordered in the AlphaFold prediction makes a figure like this not fruitful at this point. *Figure 7 et al: This will probably be edited in production, but just in case, remove the grey box outlines around the figure(s).*

**Response from authors:** We think this was an artifact of the way we inserted the figures into the Microsoft Word document. We have tried a different method and it seems to alleviate the problem on our end. Hopefully it is the case for others. Thank you.

*Reviewer #2: In this piece, Wenzel and Aquadro build on some really nice past work by further evaluating interactions between the protein-coding gene bag of marbles (bam) (in melanogaster) and endosymbiotic Wolbachia (wMel) bacteria. Briefly, bam has an important role during gametogenesis—in females bam regulates and induces daughter cell differentiation, while in males it initiates terminal differentiation of spermatogonia. Null bam mutants of both sexes are sterile, and partial loss-of-function mutants have lower female fertility. Past work has demonstrated that Wolbachia infections in females can rescue fertility reductions. While I could go on about the very interesting specific biology, this paper focuses on evaluating the specificity of the bam-Wolbachia interaction using 22 novel bam mutants. Some major results include no apparent specificity of rescue for the different functional bam regions or with predicted Bam protein structure, with rescue observed for all cases of partial fertility reduction. The authors also find no evidence that Wolbachia interacts with germline stem cells in bam mutants. These are nice findings that further develop this system.

I think this is an interesting paper, and my major comments are related to better connecting this work to relevant patterns in nature and to the possible influence of Wolbachia on bam evolution, which seems very unclear to me at this stage. I also think that including data on Wolbachia abundance (versus relying on infected/uninfected alone) would improve the interpretation of patterns. I don’t think the data support this being a general thing for “Wolbachia”, but rather a wMel-specific interaction. This is worth noting since Wolbachia and bam are diverse, and since Wolbachia infect most insects, but bam seems to be specific to Drosophila (i.e., the results are not as general as pitched).

A few bigger points:

bam evolution: The authors lay out at line 117 that rapid evolution of bam across several Drosophila motivates their interest here, with the potential for Wolbachia to have played a role. At a minimum, the authors need to summarize those patterns in more depth here. If only a handful of species have been assessed, it might be worth taking a look at new data (e.g., Suvorov et al. 2022, Current Biology). There are clades that are Wolbachia uninfected, and my thought is that with more data one could pretty quickly better test the hypothesis of a plausible Wolbachia role in rapid bam evolution. The caveat here is that Wolbachia seem to regularly switch hosts, so any uninfected species may have been infected in the past. All functional bam-Wolbachia work has been in melanogaster (I think), making this sort of analysis even more useful since we don’t know if the interaction is wMel specific. For clarity, I think that adding more context is likely to be sufficient here, but adding an additional analysis could increase impact (but is not necessary).*

**Response from authors:** We have greatly expanded on the evolutionary perspective of this interaction. This includes additional text in the introduction and in the discussion (L123 - L131). We had internally performed the analysis specifically suggested by this reviewer in this comment, but had never published the results and thank the reviewer for the encouragement to do so here. This is now at L130.  We have included the caveat mentioned about unknown past infections. (In general, we have thought about this extensively and expect a separate publication on our work in this area.)

*Also, is bam itself specific to Drosophila? I ask because I took a (very) quick look at some data, and I don’t see obvious orthologs across several holometabolous insects. This along with rapid evolution in Drosophila is really interesting, and I think other evolutionary-leaning readers will appreciate more context, even if it is redundant with prior work. I did not have time to revisit all past papers to confirm what is known (or not), and most readers won't either.*

**Response from authors:** We have added a line in the text to inform readers that *bam* is specific to the *Drosophila* genus (L125). We have ongoing work in our lab addressing this question in detail.

*Relevance of bam-Wolbachia interactions in natural populations: We know that Wolbachia must increase host fitness to initially spread in populations (Hoffman et al. 1990), and positive fecundity effects have been proposed (Fast et al. 2011, but see my comment below; Weeks et al. 2008). I think this piece could be improved by connecting the interesting genetics here to its potential role in a natural context, if there is any. As the authors know, Wolbachia are often imperfectly transmitted. This breaks up any linkage disequilibrium between host variation and Wolbachia each generation. wMel occurs at variable intermediate frequencies in melanogaster. I’m left wondering if this interaction is likely to be meaningful for Wolbachia spread in nature? Broadly, I think the piece could be improved by connecting the interesting molecular genetic results here to relevant evolutionary genetic considerations.*

**Response from authors:** Thank you for these ideas to broaden the scope of our work. We have added a section to the Discussion on population and evolutionary implications (L588+).   *Hypotheses for how Wolbachia could be rescuing bam fertility mutants: While I think the Discussion is really great and thoughtful, I want to make one relevant point that the authors might consider. The hypothesis that Wolbachia (wMau) increases the rate of GSC mitosis is less supported than indicated here. Meany et al. 2019, Evolution demonstrates a lack of any wMau fecundity effect across age and D. mauritiana backgrounds. Perhaps more importantly, this paper also demonstrates the four-fold fecundity effect reported by Fast et al. (2011) is not theoretically plausible given known parameter estimates. It’s unclear to me how the other main results of Fast et al make much sense once you consider this. I don't believe anyone has been able to replicate the Fast et al. result.*

**Response from authors:** Thank you for bringing this to our attention. We have removed the wMau findings from our manuscript and replaced it with results in *D. melanogaster* from Russell et al. 2022, which is a bioRxiv paper that was recently updated this month. In our manuscript, we specify that the Russell et al. results are preliminary, by nature of not yet being published as a peer-reviewed article (L558-L560). The hypothesis remains interesting regardless of previous publications, but it was important for us to learn of the caution around the Fast et al. 2011 results. Thank you very much.  *Wolbachia variant and titer: What Wolbachia variant is being used here (sorry if I missed this)? wMel or wMel CS? Is it a sequenced variant, and if so what wMel clade (Bergmann et al. 2012, PG)? More importantly, do the authors have any data on Wolbachia abundance in host tissues? Almost all Wolbachia effects on hosts depend on abundance/localization, and that is probably true here. The question is how much abundance varies among infected genotypes. Considering Wolbachia abundance in a quantitative way is likely to improve the interpretation of the results here, but I fully realize these data may not exist.*

**Response from authors:** This is a good point. As the *Wolbachia* field has grown, it is important to note the variants. We have clarified that we are using *w*Mel throughout the paper. We have not quantified the *Wolbachia* abundance in the tissue, though we have thought about it. I expect we would see a correlation between titer and amount of fertility rescue, as you suggest. The interpretation remains a little tricky as more wildtype-like tissue (more rescue) gives an opportunity for greater *Wolbachia* abundance (e.g., there is typically a high density of *Wolbachia* in egg chambers, so mutants wish fewer egg chambers will likely have less *Wolbachia* by nature of the tissue composition).

At present, we chose to prioritize an experimental suggestion from Reviewer 1, but appreciate this feedback and will keep it in mind for future experiments. Thank you!

*Other points:

Is there any evidence that “Wolbachia” generally interacts with bam, or are all results specific to wMel and melanogaster? If so, is all work on one wMel variant, or is it known that wMel variation contributes to variation in relevant phenotypes here? I think it is worth being clear about this. Without knowing interacting genomic factors on the Wolbachia side, it seems impossible to speculate much about the generality of this interaction from sequence analysis alone, and I don’t think other Wolbachia have been introgressed to look at phenotypes?*

**Response from authors:** We clarified this, as stated above, to say that wMel and wMelCS are known to genetically interact with *bam* (L55)*.* The *Wolbachia* in our study was all maternally inherited from the same *w*Mel infected *D. melanogaster* stock, which is stated in the manuscript L178-L179).

*Abstract: Consider being more clear about the specific Wolbachia being assessed (wMel presumably) and that the approach is using transgenics.*

**Response from authors:** Thank you! We addressed this throughout the paper, including the abstract and figures. *Line 57: It is worth noting that CI reduces egg hatch, but the strength of this reduction can vary widely (i.e., many progeny remain viable depending on the system/context; see Dylan Shropshire’s work).*

**Response from authors:** We added the information so our text is not misleading (L61, L64-65). Thank you.

*Figure 1: The authors might consider indicating patterns of selection across bam. This could easily be integrated into 1A. Presumably the focal binding regions are *relatively* conserved?*

**Response from authors:** Thank you for the suggestion to include a more evolutionary perspective. We found that adding this information to Fig 1A made it a little too busy, but we have added a supplemental figure that shows the location of divergent amino acids between *D. melanogaster* and *D. simulans* with respect to the *bam* gene (L373, Supplemental fig 5).

*Line 178: Do you happen to have egg lay, hatch, larval counts, or any other measures? Number of progeny is fine, but I’m personally interested in if you can tease apart any development-specific rescue.*

**Response from authors:** We believe we addressed this with our nurse-cell-positive egg chamber assay, in response to a comment from Reviewer 1 (see response to Line 178 comment from Reviewer 1). We note that the additional results we present correlate well with our fertility results, but some discrepancy may be present because of *bam*’s role in mitotic divisions of germ cells. At this time, we haven’t performed any developmental experiments like what was mentioned here, but we note that assessing egg lay and hatch rates would be a great experiment to do, serving as a middle ground between our original fertility assay and our new nurse-cell assay.

*Figure 2: Colors will be challenging for colorblind readers.*

**Response from authors:** Unfortunately, we need five different colors for our functional/binding regions and tried to pick colors that could be accommodating to other figures, as in figure _, where we add three new colors in. We made our immunofluorescence images colorblind friendly, but had a hard time with Figure 2. *Figure 2: What is the interpretation of fertility enhancement relative to controls in multiple cases? Maybe there isn’t one.*

**Response from authors:** This is a great question and a puzzle for us, too. Because of *bam*’s intrinsically disordered nature and multiple functional partners, perhaps as mutations can be disruptive to Bam function, a few may also enhance Bam function.  *Line 343: Wow. I think a 20-fold increase is striking and could rise to the level of being noted in the abstract.*

**Response from authors:** Thank you for this suggestion! We have added it to our abstract (L17)

*Figure 4: It seems plausible that Wolbachia abundance varies among infected genotypes, and if the authors have those data, it would be good to know if the degree of recovery is a function of variable Wolbachia abundance in tissues or even whole bodies.*

**Response from authors:** This comment addresses the same point as an earlier comment by this same reviewer that is entitled “Wolbachia variant and titer.” Please see our response to that comment. *Line 377: Or variation in Wolbachia abundance.*

**Response from authors:** We added this (L452). Thank you!

*Line 473: This result cannot be replicated as noted above.*

**Response from authors:** Thank you. We addressed this as noted above.
